# Permeability versus Design in TPMS Scaffolds

**DOI:** 10.3390/ma12081313

**Published:** 2019-04-22

**Authors:** A. P. G. Castro, T. Pires, J. E. Santos, B. P. Gouveia, P. R. Fernandes

**Affiliations:** IDMEC, Instituto Superior Técnico, Universidade de Lisboa, 1649-004 Lisbon, Portugal; tiago.a.h.v.pires@tecnico.ulisboa.pt (T.P.); jemidiocsantos@gmail.com (J.E.S.); bgouveia@tecnico.ulisboa.pt (B.P.G.); paulo.rui.fernandes@tecnico.ulisboa.pt (P.R.F.)

**Keywords:** bone scaffolds, TPMS, tissue engineering, numerical modeling, biomechanics, permeability

## Abstract

Scaffolds for bone tissue engineering are porous structures that serve as support for cellular growth and, therefore, new tissue formation. The present work assessed the influence of the porous architecture of triply periodic minimal surface (TPMS) scaffolds on their macroscopic permeability behavior, combining numerical and experimental methods. The TPMS scaffolds considered were Schwartz D, Schwartz P, and Gyroid, which have been previously studied for bone tissue engineering, with 70% porosity. On the experimental side, these scaffolds were produced by MultiJet 3D printing and tested for fluid passage to calculate their permeability through Darcy’s Law. On the numerical side, finite element (FE) models of the scaffolds were simulated on ABAQUS^®^ for fluid passage under compression to assess potential fluid concentration spots. The outcomes revealed that the design of the unit cell had a noticeable effect on both calculated permeability and FE computed fluid flow velocity, regardless of the identical porosity, with the Gyroid scaffold having higher permeability and the Schwartz P a lower probability of fluid trapping. Schwartz D had the worst outcomes in both testing modalities, so these scaffolds would most likely be the last choice for promoting cell differentiation onto bone cells. Gyroid and Schwartz P would be up for selection depending on the application and targeted bone tissue.

## 1. Introduction

Scaffolds can be defined as porous structures that act as support for cell viability, attachment, proliferation and homing, osteogenic differentiation, vascularization, host integration, and load bearing. They permit the diffusion of oxygen, nutrients, and metabolic waste, as they are tailored to ensure adequate cellular growth and proliferation on the targeted tissue [1]. Thus, the characteristics of scaffolds can be modulated towards bone tissue engineering (BTE) applications in four main vectors: biological requirements, structural features, biomaterial composition, and fabrication process. In the specific case of BTE, structural integrity for load bearing is one of the key aspects for enhancing bone shape and function during and after the regeneration and remodeling processes [2]. In addition, with the advent of 3D printing, it is an advantage if scaffolds can be manufactured through this technology. 3D MultiJet printing has proven to be a good option for scaffold manufacture, benefiting from efficient cost control and high production accuracy [3]. 

For all that to happen, scaffolds should have distributed, interconnected pores and display high porosity in order to ensure cell penetration, vascular ingrowth, nutrient diffusion, and waste product elimination. Scaffolds crafted through the triply periodic minimal surface (TPMS) method meet these requirements. TPMSs are defined mathematically as infinite and periodic surface curvatures [4]. This method targets the development of scaffolds with the optimal relation between levels of porosity and stiffness to promote different cellular growth rates [5], as they allow for fully controllable homogenous BTE scaffold projects, parting from the design of the repeatable unit cell [6].

Studies have found that cell growth into a scaffold depends on how well nutrients can permeate through the porous structure during the cell culture process [7,8,9]. Therefore, good permeability of the scaffold, translated to the properties of the structure, means the right pore size distribution, high pore interconnectivity, and sufficient porosity [7,8,9]. Furthermore, permeability affects the magnitude of pressure and shear forces inside scaffolds, identified as potential stimuli for cellular differentiation or functional adaptation, for cell seeding efficiency and in vivo new tissue formation [10,11,12].

This work deals with the analysis of permeability as a function of geometry of TPMS scaffolds, combining numerical and experimental methods. On the one hand, the permeability of 3D printed scaffolds was evaluated by measuring the pressure drop through the sample [9]. On the other hand, finite element (FE) simulations were performed to assess not only the potential fluid concentration spots inside the scaffold structure (and associated cell substrate) but also the profile of fluid flow velocity within this structure [11]. The combination of these techniques allowed for understanding these scaffolds’ biomechanical behavior in terms of permitting sufficient fluid flow for BTE applications, as well as the relationship between their basic unit cell geometry and the measured permeability.

## 2. Materials and Methods

Different scaffolds have been designed based on the TPMS method [13]. This work made use of the Schwartz D, Gyroid, and Schwartz P models with 70% porosity, which are hereinafter referred to as SD70, SG70, and SP70, respectively. The selection of the porosity level was based on the relationship between 3D printing efficiency and ensuring a convenient fluid flow, since this is one of the main requirements for cellular growth [6,8,14]. The basic cubic unit of each scaffold was created in STL format with a custom TPMS generator [6,15] and then repeated according to the application.

### 2.1. 3D Printing and Experiments

**Figure 1:**
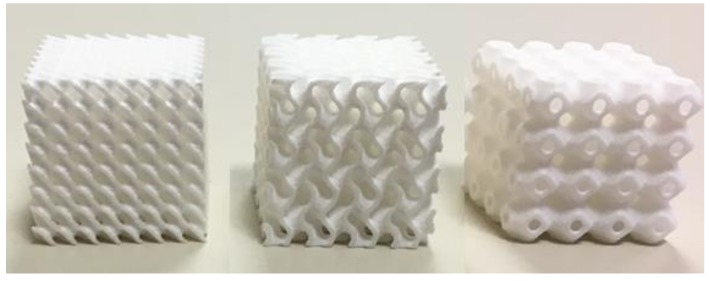
3D printed scaffolds: Schwartz D (SD70), Gyroid (SG70), and Schwartz P (SP70) (from left to right).

The scaffold samples tested in the present work (Figure 1) were printed in the Tissue Biomechanics Lab of Instituto Superior Técnico (Lisbon, Portugal) using the MJP 3600^®^ MultiJet printer (3D Systems, Rock Hill, SC, USA) and the commercial material Visijet M3 Crystal (recommended by the printer’s manufacturer) [16]. This material presents a tabled Young’s modulus value of 1.46 GPa and it is certified with USP Class VI norm, allowing its use in several medical applications [16]. After designing the geometries, the scaffolds were printed in 13-mm sided cubes (requisite for the permeability apparatus, to be discussed next), corresponding to 4 × 4 × 4 basic cubic units of 3.25-mm sides. Three samples were printed per design.

It is important to note that for using MultiJet techniques, the printed geometries should consider paths large enough to allow for the draining of support wax from the interior of the scaffold, which was first obtained by heating at 60 °C for at least 48 h. The initial protocol suggested by the manufacturer indicated a minimum of 8 h, but treatment tests have shown that the weight reduction corresponding to the expected complete wax removal could only be effective after a longer period. This treatment was finalized with an isopropanol bath. The validation of the wax removal was made by comparing the weights of each specimen along the treatment time with the weight of a compact solid with the same dimensions.

**Figure 2:**
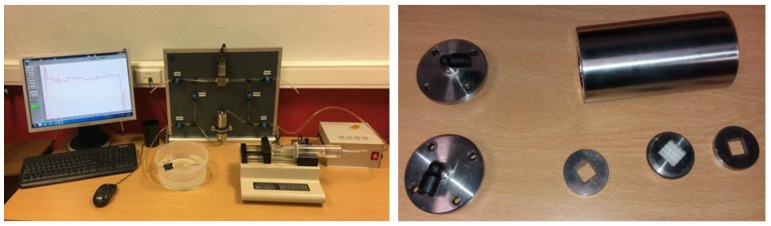
Experimental apparatus for the permeability tests (left: overview; right: permeability chamber parts).

Having completed the fabrication and treatment of the scaffolds, they were placed, one at a time, in the experimental apparatus for the permeability tests shown in Figure 2. The apparatus consisted of a syringe mounted in a machine that allowed a controlled constant flow rate, a cubic sample chamber with 13-mm sides, and a sensor responsible for measuring the pressure drop (i.e., the water pressure before and after the fluid permeated the scaffold). Before conducting each test, the system was purged of all air. The tests were conducted with increasing flow rate steps of 20 mL/min, from 20 to 100 mL/min. The limit in flow rate was related to the measurement range of the pressure sensor. The measured pressure drop (Δ*P*) was then used to calculate the permeability (*k*) of the sample, which was given by Darcy’s law (Equation (1)). *Q* is the fluid rate, *μ* is the dynamic viscosity of the fluid, *L* is the length of the sample, and *A* is the cross-sectional area of the sample [17].

(1) $$k\;=\;\frac{Q\mu L}{A\Delta P}$$

### 2.2. FE Modeling and Simulation

**Figure 3:**
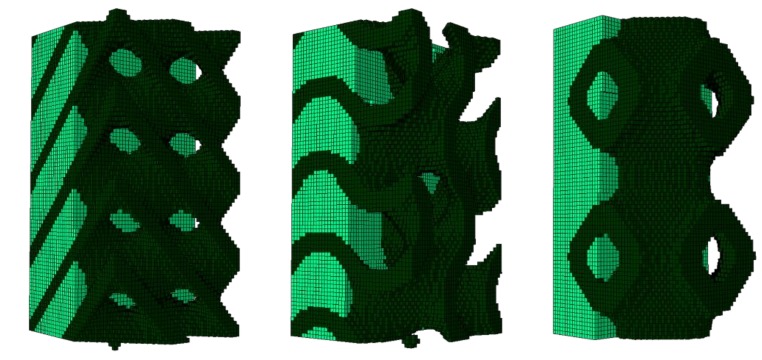
Finite element (FE) models of triply periodic minimal surface (TPMS) scaffolds embedded in 0.20% collagen substrate (left: SD70; center: SG70; right: SP70). Please note that dark green represents the scaffold and light green the collagen substrate.

From the numerical point of view, it was not feasible to use cubic scaffolds with 13-mm sides. The FE models of the scaffolds were paralepidids with a 1 × 1-mm cross section and 2-mm height for the sake of reducing the computational weight while ensuring a preferential uniaxial compression direction (vertical). Scaffold pores were filled with 0.20% collagen hydrogel, following the method described by Castro and Lacroix [11] (hyperporoelastic material with strain-dependent permeability). Quadratic eight-node hexahedral elements (C3D8RPH) were used to comply with the poroelastic behavior of collagen. Figure 3 shows the complete FE models (scaffold plus collagen). Each one of the three FE models was built with 136,161 nodes and 128,000 elements, since they all share the same basic cubic structure. However, Table 1 details the number of scaffold and collagen elements per model, since the final structure was different despite having the same porosity.

The asymptotic homogenization method described by Guedes and Kikuchi [18] allowed the calculation of the equivalent elastic coefficients for periodic porous structures (Table 2), used as material inputs for the scaffolds on the models (linear elastic material). This approach has been previously validated [6]. The time-dependent FE simulations were run on ABAQUS^®^ (Dassault Systèmes Simulia Corp., Johnston, RI, USA) and consisted of ramp 8% confined vertical compression imposed on the top of the scaffold for 10 s [11,19]. Fluid flow velocity distribution at the end of the test was analyzed to evaluate the potential fluid concentration spots along the internal structure of each scaffold.

## 3. Results

**Figure 4:**
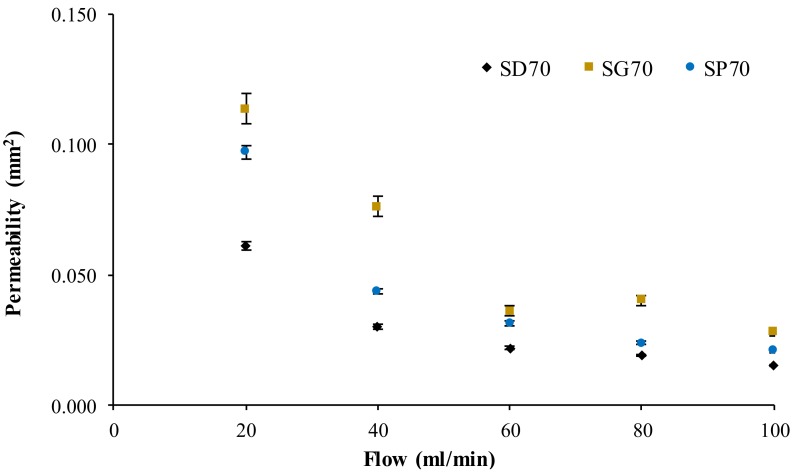
Calculated permeability for the TPMS scaffolds as a function of the flow rate, including the experimental standard deviation.

Figure 4 shows the average calculated permeability for the three scaffold models as a function of the increasing flow rate. It can be observed that the permeability variation was reduced above the flow rate of 60 mL/min for all the models. The maximum standard deviation in the experiments was 7.5%, and the average standard deviation was 3.5%.

**Figure 5:**
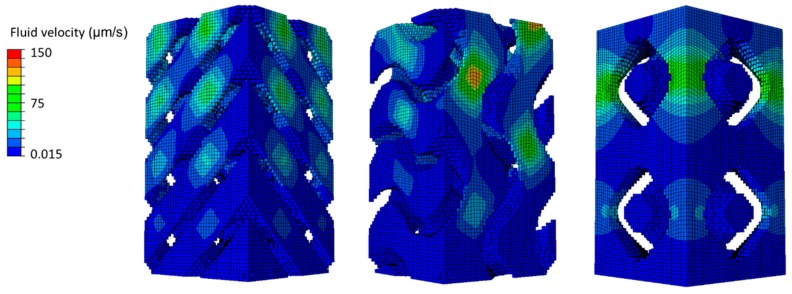
Fluid flow velocity distribution at peak compression over the collagen substrate in contact with TPMS scaffolds (left: SD70; center: SG70; right: SP70). Please note that the scaffold has been removed for the sake of visualization.

**Figure 6:**
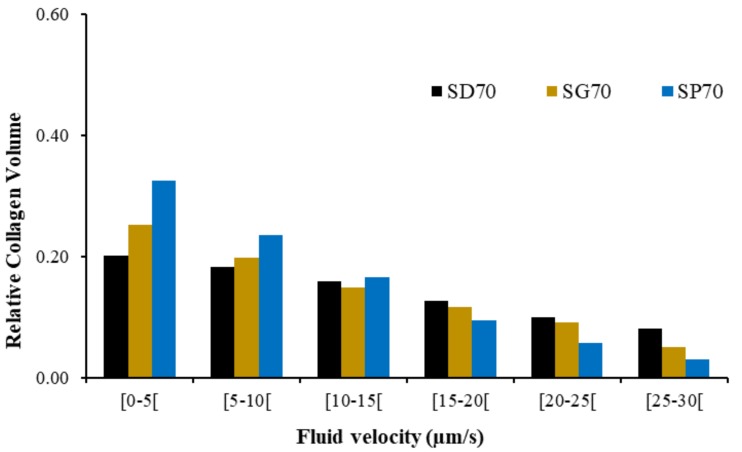
Fluid flow velocity distribution (µm/s) in the collagen substrate elements versus their relative volume, at peak compression, for the three FE models.

After characterizing the permeability of the TPMS scaffolds, Figure 5 shows the fluid flow velocity distribution at peak compression over the collagen substrate, on the FE simulations, for the three scaffold FE models. Figure 6 summarizes the fluid flow velocity distribution in respect to the percentage of collagen volume, also at peak compression. It is visible that the fluid flow distribution patterns varied with the geometry of the scaffold and also that the magnitude (and range) of velocities were related in the three models.

## 4. Discussion

The experimental permeability outcomes shown in Figure 4 follow what was theoretically expected, that is, higher flow rates resulted in lower permeability. The average difference between the permeability calculated for SG70 and SP70 was 27%, while the analogous measure for SG70 and SD70 was 49%, meaning that the higher and lower permeabilities corresponded to SG70 and SD70, respectively, throughout the range of studied flow rates. The average experimental permeability of the SG70 samples at 60 and 80 mL/min were the only points outside of the expected distribution (i.e., permeability would theoretically be higher at 60 mL/min or lower at 80 mL/min).

There was no linear relationship between the homogenized equivalent elastic coefficients and the calculated permeability. SD70 was the stiffer structure and it presented the lower permeability, but this was not true for SG70 and SP70, as this last one had the lowest Young’s modulus but not the highest permeability. The maximum difference in Young’s modulus (SD70 to SP70) was limited to 40%. Hence, permeability seemed to be mostly influenced by the microstructure of each scaffold, instead of porosity (which was the same for all the scaffolds here). This suggests that the choice of the structure would have to be application dependent, and even the equivalent stiffness needs to be evaluated case-by-case.

Following this line, the FE simulations revealed that the interaction between scaffold and collagen substrate could be indicated for cell differentiation towards bone formation [11,20], particularly in the internal layers, as Lacroix and Prendergast [20] (and later Castro and Lacroix [11]) suggested that ideal fluid velocity for bone cell differentiation should be under 3 μm/s, and also that cell death could start above 20 μm/s. The fluid flow velocity concentration peaks seen in Figure 5 were mostly located on the superior and outside layers, which was probably related to the applied load and boundary conditions. These are then good indicators that would have to be confirmed in cell-seeding experiments. In detail, SP70 seems to have the more evenly distributed spots of fluid flow concentration. In addition, Figure 6 suggests that lower fluid flow velocities will be more evenly distributed for the substrate around SP70 (73% of the collagen elements with velocities under 15 μm/s against 54% for SD70 and 60% for SG70). SD70 and SG70 registered the same 85% collagen elements with velocities under 30 μm/s, while SP70 registered a slightly higher value of 91%.

Summarizing all the outcomes, one can argue that the design of SP70 allows for better identification of the potentially harmful (for cell differentiation and proliferation) fluid flow concentration spots, even if this does not correspond to having the highest permeability. However, SD70 had the lowest permeability and a less favorable fluid flow velocity distribution, which suggests that this design, at least for this porosity level, is the worst choice for BTE applications among the three analyzed designs. Finally, the SG70 model, given its intermediate outcomes, would be considered together with SP70.

The limitations of the study are identified in the number of samples in the permeability experiments, which will have to be increased in further investigations. Still, three samples were considered enough given the complexity of each experiment, the low standard deviation obtained, and the costs associated with sample production for this early study. The need for reducing the FE models into two basic cubic units is also an issue to be addressed in future studies, while the formation of 4 × 4 × 4 basic cubic units could also be extended (e.g., 8 × 8 × 8 or 10 × 10 × 10) to verify the independence of the permeability (and other metrics) from the number of basic cubic units.

## 5. Conclusions

This work was able to characterize the permeability and potential internal fluid flow distribution of three different TPMS scaffolds. It was found that there is no direct relationship between structural stiffness, permeability, and fluid flow distribution—at least for these three models. However, it can be concluded that choosing a given porosity according to the target organ (bone, in this case) is not enough to differentiate (or select) a given scaffold design. In fact, scaffold design will play a major role both in relation to the tissue and the substrate material where cells will be seeded for differentiation and proliferation. 

In this study, the Schwartz D architecture proved to be less favorable for BTE applications than Schwartz P or Gyroid. However, different porosity levels and a wider range of flow rates may be considered in future works to complete and verify the current data in order to create a methodology for application-dependent selection of scaffolds in BTE. 

## Figures and Tables

**Table 1:** Number of elements per material.

Model	Scaffold Elements	Collagen Elements
SD70	46,372	81,628
SG70	46,024	81,976
SP70	42,512	85,488

**Table 2:** Young’s modulus (E) of the TPMS scaffolds calculated by the homogenization method of Guedes and Kikuchi [18].

Model	E (MPa)
SD70	171.37
SG70	145.05
SP70	103.54
